# Management of Black Root Disease-Causing Fungus Fusarium solani CRP1 by Endophytic Bacillus siamensis CNE6 through Its Metabolites and Activation of Plant Defense Genes

**DOI:** 10.1128/spectrum.03082-22

**Published:** 2023-02-06

**Authors:** Pralay Shankar Gorai, Ranjan Ghosh, Suvranil Ghosh, Smriti Samanta, Animesh Sen, Suraj Panja, Surendra Kumar Gond, Narayan Chandra Mandal

**Affiliations:** a Mycology and Plant Pathology Laboratory, Department of Botany, Visva-Bharati, Santiniketan, India; b Department of Botany, Bankura Sammilani College, Bankura, India; c Division of Molecular Medicine, Department of Biochemistry, Bose Institute, Kolkata, India; d Regional Ayurveda Research Institute, Gangtok, Sikkim, India; e Rice Biotechnology Laboratory, Department of Biotechnology, Visva-Bharati, Santiniketan, India; f Department of Botany, MMV, Banaras Hindu University, Varanasi, India; USDA—San Joaquin Valley Agricultural Sciences Center

**Keywords:** black root rot disease, biofilm inhibition, biocontrol, *Cicer arietinum* L., *Fusarium solani* CRP1, host defense genes, *in vivo* experiment, molecular docking, secondary metabolites

## Abstract

Black root rot disease of Cicer arietinum L. is accountable for substantial loss in chickpea production worldwide. Endophytic Bacillus siamensis CNE6 has previously shown multifaceted plant growth-promoting, broad-spectrum antifungal, and chickpea plant-colonizing potential. In the present study, the strain Bacillus siamensis CNE6 was used for controlling black root rot disease caused by Fusarium solani CRP1 in chickpea. CNE6 showed strong antagonistic potential against CRP1 both *in vivo* and *in vitro*. Scanning electron microscopic studies indicated cellular deformation of CRP1 due to production of β-glucanase, protease, and other secondary metabolites. A total of five compounds were detected from the cell-free supernatant (CFS) of the ethyl acetate (EA) fraction of CNE6 through gas chromatography-mass spectrometry analysis. A confocal microscopic study demonstrated strong inhibition of biofilm formation of the pathogen CRP1 by the EA fraction of CFS of CNE6. Molecular docking analysis revealed that one compound, (2E)-6-methoxy-2-[(4-methoxyphenyl)methylidene]-2,3-dihydro-1-benzofuran-3-one, may inhibit the activity of lanosterol 14-alpha demethylase, which is involved in ergosterol biosynthesis and beta-tubulin assembling. *In vivo* experiments also showed the efficacy of CNE6 for increasing chickpea growth as well as upregulation of four defense genes (*CHI1*, *PAMP*, *PR2B*, and *TF1082*) upon pathogenic challenge. Thus, our results strongly suggest a positive role for CNE6 as a prospective biocontrol agent for combating Fusarium solani in chickpea.

**IMPORTANCE** The present work was undertaken to explore an effective biocontrol agent against the destructive black root rot disease of chickpea. We have used an efficient bacterial endophyte, CNE6, which can colonize in the chickpea root system, produce secondary metabolites and enzymes to degrade pathogenic cellular integrity, inhibit pathogenic establishment by rupturing biofilm formation, and induce host immunity upon treatment. Interaction of the bacterial metabolite was also observed with lanosterol 14-alpha demethylase, which is an important component in fungal membrane functioning. Being an endophyte, Bacillus siamensis CNE6 fulfills a suitable criterion as a biocontrol agent to control black root rot disease in chickpea and has huge prospects for use commercially.

## INTRODUCTION

Legumes belong to the family Fabaceae and are considered important crops especially due to their high protein content. Among the different edible legumes, chickpea (Cicer arietinum L.) is considered one of the most important annual crops and plays a significant role in food security for the population of India and Sub-Saharan Africa by fulfilling the protein scarcity of daily food ration (1). In addition to different abiotic factors, a significant loss of chickpea yield also occurs due to attacks by various pathogens, such as bacteria, fungi, virus, nematodes, and insects, etc. A major loss of chickpea production was found globally due to attacks by fungal pathogens, including *Ascochyta* blight, Fusarium wilt, Fusarium black root rot, *Rhizoctonia* dry and wet root rot, *Sclerotium* collar rot, and *Cercospora* leaf spot (2). Among fungal diseases, black root rot disease caused by the soilborne fungal pathogen Fusarium solani is one of the most devastating factors causing massive loss in chickpea production (3, 4).

Although there are various agrochemicals available on the market that have a significant role for plant disease management and to increase crop yield, extensive utilization of chemicals in the fields has deleterious effects on humans as well as the environment. Due to their high toxicity, a large number of synthetic fungicides have also been banned in the Western world (5).

For the development of sustainable agriculture through less use of chemicals, scientists are trying to develop alternative strategies for controlling plant diseases in an eco-friendly manner. Among such strategies, the use of biocontrol agents which can suppress diseases of plants without impacting the environment and human health is considered a suitable approach. Many scientists have applied nonendophytic biocontrol agents like rhizospheric and phylospheric organisms for suppressing plant diseases. Recently, endophytic microorganisms have been preferred over nonendophytic microorganisms by researchers as biocontrol agents in agricultural fields because endophytes can take shelter and colonize within host plant tissues very easily and they remain protected their entire life span. After colonizing within host tissues (6), they show outstanding performance in overcoming both biotic and abiotic stresses faced by the host plant in the natural environment. Endophytic biocontrol organisms can suppress phytopathogens by producing different antagonistic metabolites, such as hydrolytic enzymes (7, 8), lipopeptides (9), and other volatile compounds (10). Several genera of endophytic bacteria, such as *Bacillus*, *Strenotrophomonas*, Pseudomonas, *Burkholderia*, Enterobacter, *Micrococcus*, and *Serratia*, have been reported previously for the synthesis of lytic enzymes, including chitinase, protease, and β-glucanase, which are responsible for fungal cell wall degradation during antagonistic interactions (11–13). Endophytic bacterial genera like Pseudomonas and *Strenotrophomonas* derived from *Datura metel* give bioprotection against the wilt pathogen of tomato Fusarium oxysporum f. sp. *lycopersici* by producing lytic enzymes such as protease, chitinase, and pectinase (14). Endophytic *Bacillus* spp. isolated from wild solanaceous species can control Fusarium wilt disease in tomato by producing lipopeptide antibiotics, expressing chitinase genes, and stimulating host plant defense genes (15). Scientists have also explored that some endophytic bacteria bear several biocontrol genes which are expressed for producing different compounds like pyrrolnitrin (PRN), phenazine, pyoluteorin, phenazine-1-carboxylic acid, hydrogen cyanide (HCN), and 2,4-diacetylphloroglucinol and play a pivotal role in suppressing pathogen growth (16). Endophytic bacteria recovered from Vicia faba, including Pseudomonas yamanorum B12, Pseudomonas fluorescens B8P, and Rahnella aquatilis B16C, have the ability to produce siderophores, HCN, and PRN (17). Among these, both HCN and PRN compounds have antagonistic activity against several soilborne fungal pathogens (18, 19). Alternatively, some endophytes can indirectly improve the host defense by inducing the systemic resistance of plants against phytopathogens (9, 20, 21). Endophytic Bacillus amyloliquefaciens subsp. *plantarum* strain SV65 can produce the lipopeptide fengycin, which can activate induced systemic resistance (ISR) in tomato (15). The endophyte Bacillus subtilis strain SGJW03 can also upregulate genes, namely, the PR-1, PR-4, and SOD-2 genes of maize, but the extracted lipopeptide from this strain alone did not show any such type of host gene expression (9). Both of these endophytes were able to inhibit the phytopathogen Fusarium.

To increase chickpea production and to avoid the use of chemical fungicides, the main objective of the present study was to control black root rot disease of chickpea by using the endophytic biocontrol organism Bacillus siamensis CNE6, which was previously isolated in our laboratory from the nodules of healthy chickpea and reported to have sufficient antifungal potential (22). By considering this, the specific objectives of the present studies were the following: (i) isolate the pathogen causing black root rot disease of Cicer arietinum L. from an infected field of the Birbhum district, West Bengal, India, and evaluate the antagonistic activity of CNE6 against the isolated pathogen, (ii) study the probable mechanisms of antagonism and characterization of antifungal metabolites produced by strain CNE6, and (iii) check the efficacy of CNE6 for inducing the chickpea defense system against pathogenic challenge as well as for enhancing overall plant growth. The present finding hypothesized that the endophytic strain CNE6 has great potential for controlling the black root rot disease-causing pathogen Fusarium solani CRP1 of chickpea by producing hydrolytic enzymes and different antifungal metabolites as well as by inducing the host defense system.

## RESULTS

### Isolation, characterization, and identification of black root rot pathogen.

Initially, from the infected roots of *Cicer arietinum* L. with black root rot symptoms, five fungal colonies were isolated and purified. All of the isolated strains showed similar colony morphologies such as circular, smooth, white cottony mycelium on malt extract agar plates. Light microscopic observations also suggested that all these isolates can produce round to oval microconidia, 3- to 5-celled sickle-shaped macroconidia, and septate mycelial structures. Based on the morphological characteristics, the pathogens were preliminarily identified as species of Fusarium.

**FIG 1:**
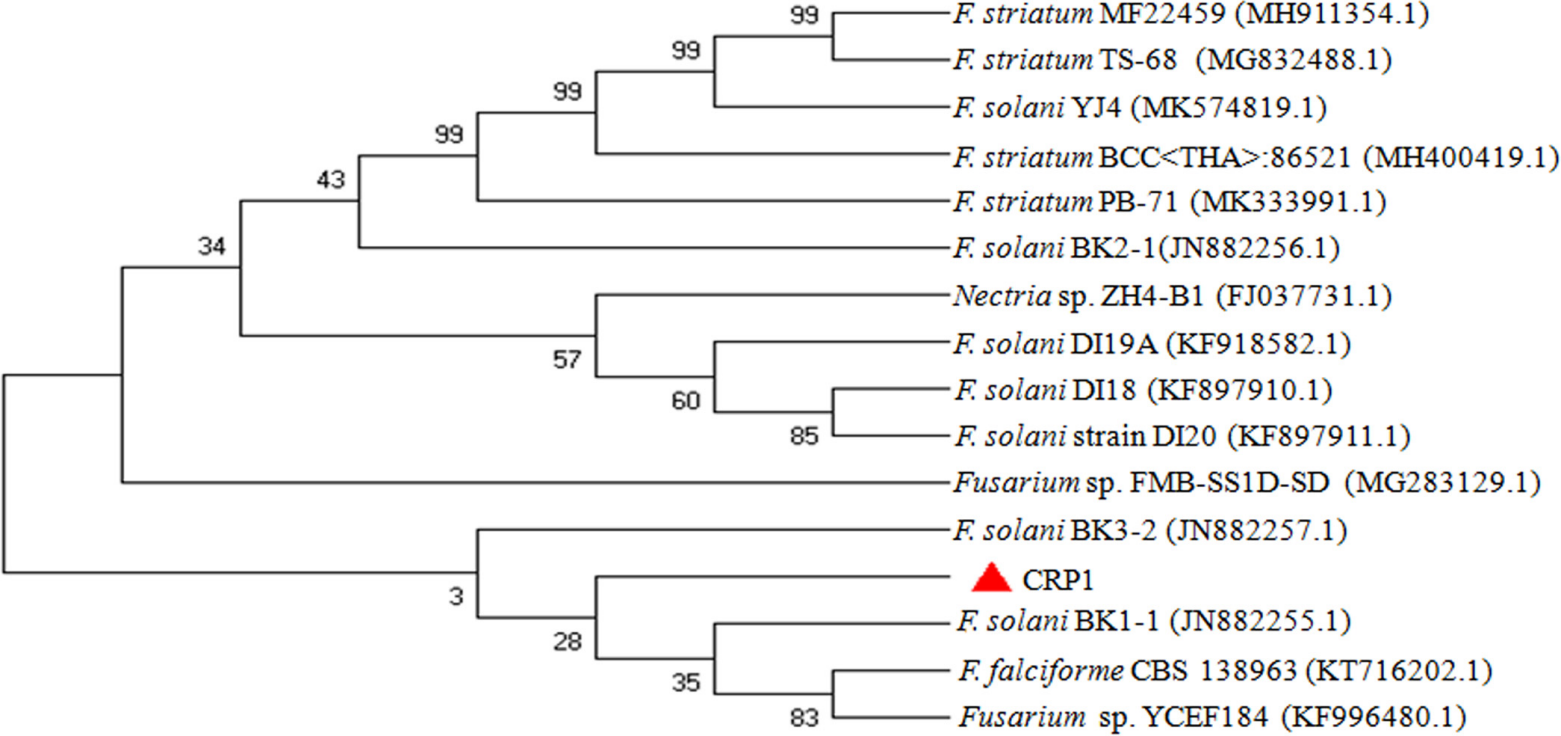
Characterization and identification of isolated pathogen. A neighbor joining phylogenetic tree of isolated pathogen CRP1 was constructed using nucleotide sequence of the ITS region of rRNA.

Among the isolated pathogenic strains, strain CRP1 was taken as a representative for further study, and therefore it was identified based on the sequencing of internal transcribed spacer (ITS) regions. Amplified products of the ITS region of rRNA gene produced a single discrete band (~600 bp) on a 1.2% agarose gel. The 538-bp nucleotide sequence of isolated pathogen CRP1 exhibited 100% pairwise similarity with Fusarium solani clone BK1-1 (GenBank accession no. JN882255.1). A neighbor joining phylogenetic tree of CRP1 also showed a close relationship with F. solani (Fig. 1).

### Confirmation of CRP1 as black root rot pathogen of chickpea.

**FIG 2:**
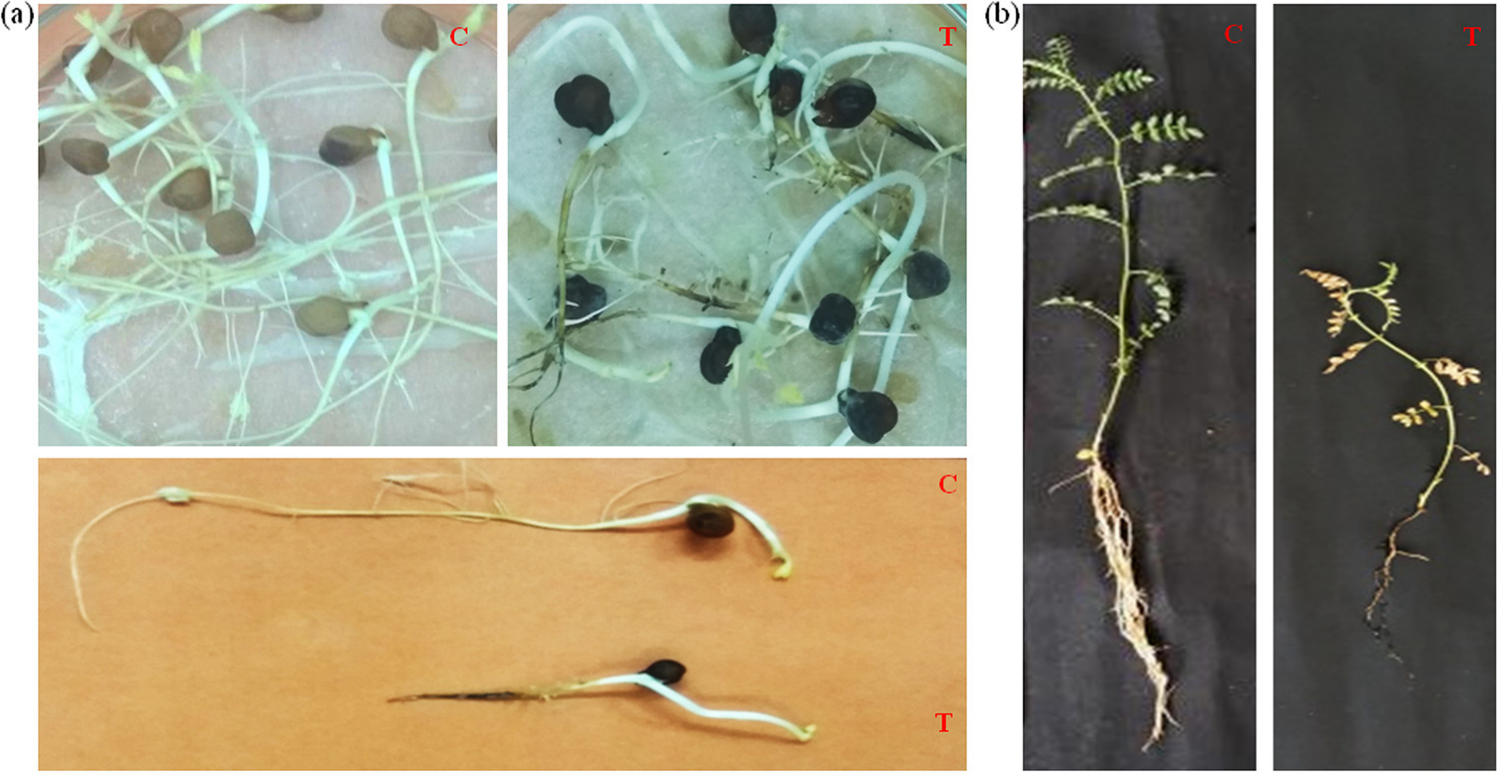
Pathogenecity test of isolated pathogen CRP1 in chickpea plants. (a) *In vitro* laboratory assessment; (b) *in vivo* pot experiment. C, untreated control; T, treated with CRP1 (*n* = 3).

In both *in vivo* and *in vitro* pathogenicity studies, chickpea plants treated with the pathogen CRP1 were affected and symptoms were produced, especially in the root regions. Treated chickpea roots turned black in color (Fig. 2), which indicated the development of black root rot disease, while no such color was found to develop in the roots of untreated control plants. In the *in vivo* experiment of the pathogenicity test, chickpea plants exhibited yellow leaves and a rotten root system along with maximally infected finer roots, and the remaining roots looked black, closely similar to the black root rot disease symptoms noted during the sample collection from infected fields. But in untreated control chickpea plants, no such symptoms were observed. From the infected plants, again the pathogen was reisolated by following the same method and again identified as F. solani by sequencing of the ITS region.

### *In vitro* antagonistic activity of *B. siamensis* CNE6 against F. solani CRP1.

**FIG 3:**
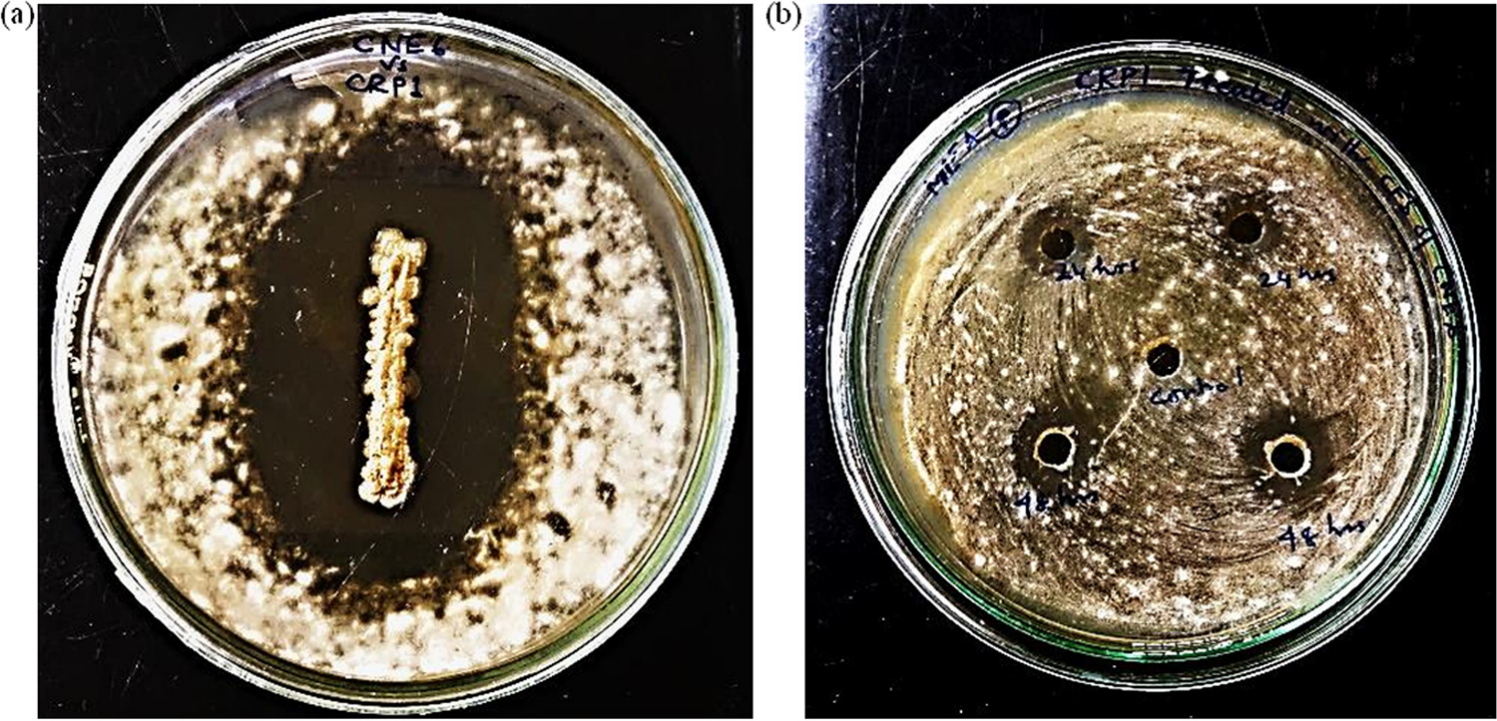
Zones of inhibition produced by *B. siamensis* CNE6 against isolated pathogen F. solani CRP1. (a) Dual culture overlay assay; (b) agar well diffusion assay (upper two wells, 24-h-grown CFS; lower two wells, 48-h-grown CFS; middle well, untreated control). Values are expressed as the mean ± SD (*n* = 3).

The isolated endophytic bacterial strain CNE6 significantly inhibited the growth of the pathogen CRP1, as observed in both dual culture overlay and agar well diffusion assays. Zones of nearly 35 ± 1.0 mm were visible surrounding the bacterial colonies in dual culture overlay plates (Fig. 3a). In the agar well diffusion assay, cell-free supernatant (CFS) of both 24-h- and 48-h-grown bacterial cultures produced inhibition zones against the pathogen CRP1 (Fig. 3b). An increase in the zone diameter (16.5 ± 1.5 mm) was also observed for 48-h-grown CFS in comparison to that of the 24-h-grown culture.

### Inhibition of radial growth of CRP1 by CFS of CNE6.

**FIG 4:**
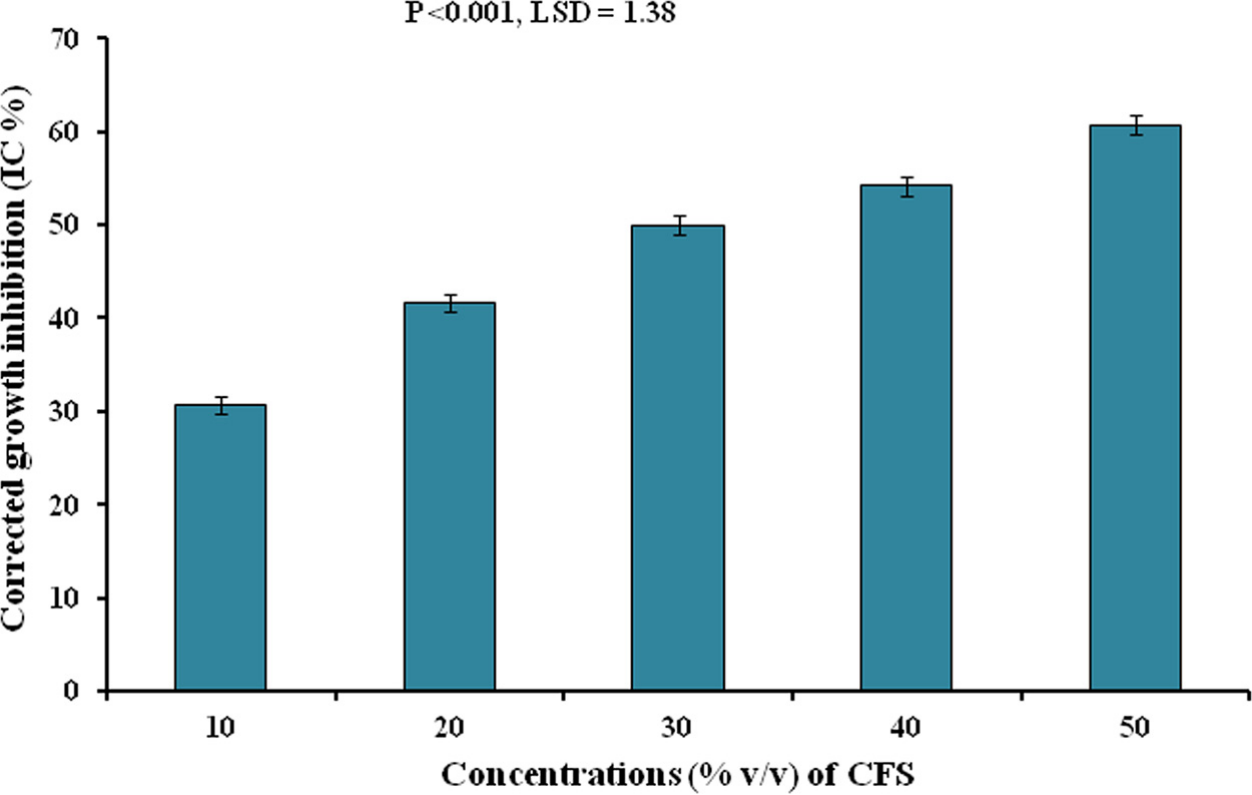
Corrected growth inhibition of F. solani CRP1 at different concentrations (%, vol/vol) of CFS of *B. siamensis* CNE6. Values are expressed as the mean ± SD (*n* = 3). Significance level, *P* < 0.001. LSD, Fisher’s least significant differences.

Measurement of fungal radial growth is a very popular but indirect measure for assessing antifungal properties of any chemical or metabolite (23). The CFS of CNE6 was found effective for significant (*P* < 0.001, least significant difference [LSD] = 1.38) inhibition of the radial growth of the pathogenic fungus CRP1 (Fig. 4). The colony diameters were markedly reduced with increased concentrations of CFS. In the presence of 50% CFS, 60.70% ± 1.10% inhibition in the radial growth of CRP1 was observed.

### Effects of CFS of CNE6 on conidial germination of CRP1.

The CFS of CNE6 was also found effective for inhibiting the conidial germination of the isolated pathogen CRP1. Significant decreases in conidial germination were observed along with increased concentrations of bacterial CFS. At 50% CFS, only 17.5% of the conidia were able to germinate (Table 1).

**TABLE 1:** Percentages of germination of CRP1 conidia upon treatment with different concentrations of CFS from CNE6^*a*^

Concn of bacterial CFS (%)	Germinated conidia (%)
0	93.62 (±2.19) A
10	84.56 (±2.7) B
20	52.30 (±1.91) C
30	38.10 (±2.50) C
40	26.78 (±3) C
50	17.5 (±1.33) C

a An unpaired t test, one per row, was performed where the P was <0.05. Values are means ± SD (n = 3). Values followed by different letters are significantly different.

### SEM observation.

**FIG 5:**
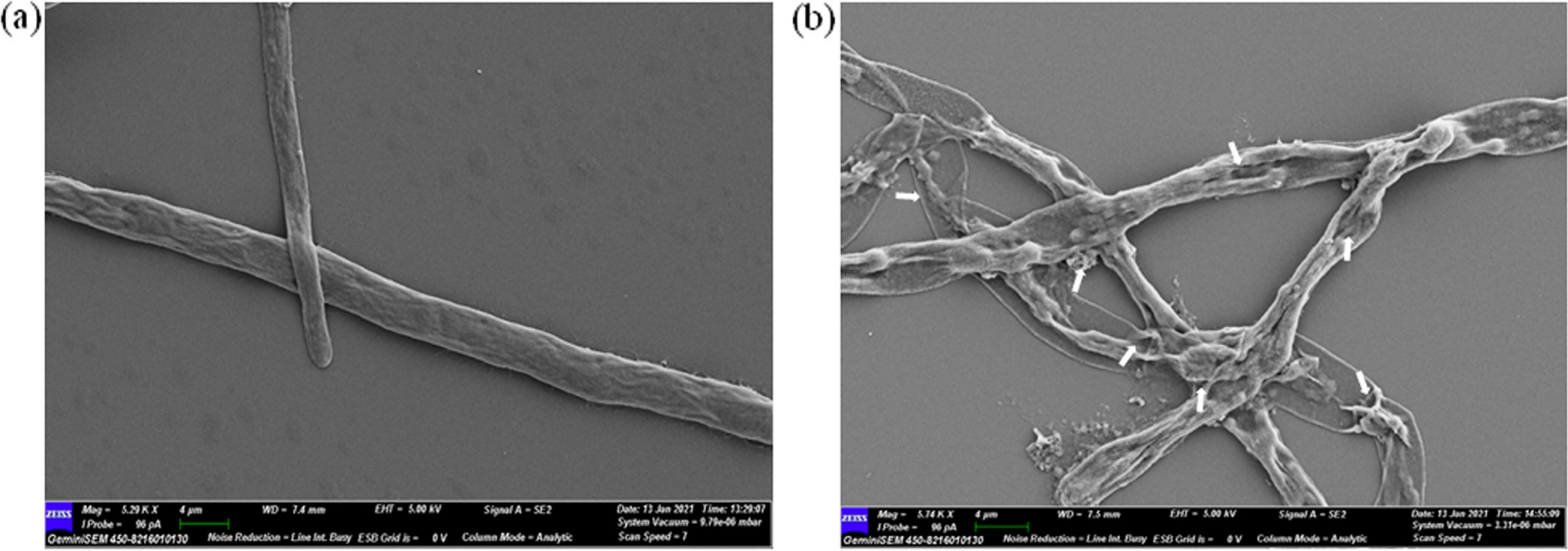
SEM observation of morphological deformations of fungal phytopathogen caused by *B. siamensis* CNE6. (a) Untreated control; (b) treated with CNE6. (Micrographs with the highest clarity were selected from several randomly taken micrographs.)

Antifungal metabolites secreted by strain CNE6 have a prominent visible effect on the mycelial structures of black root rot pathogen F. solani CRP1, as observed by a scanning electron microscopic (SEM) study. The cell wall of the pathogenic fungus CRP1 was degraded abruptly upon treatment with CNE6 (Fig. 5b). In the case of the control, intact mycelial structures were found (Fig. 5a).

### Nature of antifungal metabolites of CNE6.

**FIG 6:**
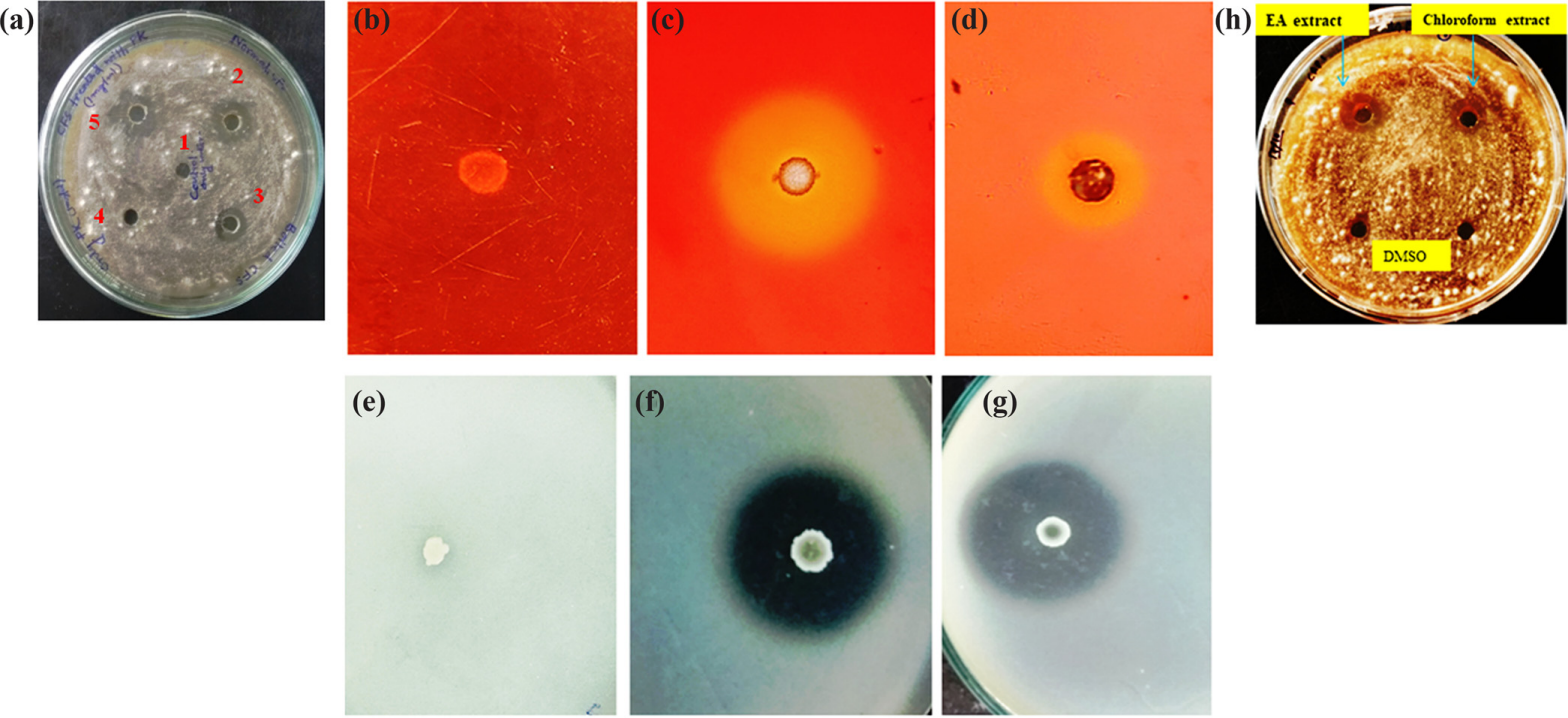
Characterization of antifungal metabolites produced by CNE6. (a) Determination of the nature of antifungal metabolites produced by CNE6 against the pathogen CRP1. Well numbers signify the following: 1, control; 2, normal CFS; 3, boiled CFS; 4, PK only; 5, CFS treated with PK. (b to d) Determination of β-glucanase production on a 1% laminarin-containing plate. (b) Negative control (laboratory isolate, unidentified strain CNE3); (c) halo zone produced by CNE6 indicating β-glucanase production; (d) positive control (Bacillus velezensis SEB1). (e to g) Determination of protease production on peptone gelatin agar plate. (e) Negative control (strain CNE3); (f) halo zone produced by CNE6 indicating protease production; (g) positive control (Bacillus velezensis SEB1); (h) zone produced by crude antifungal metabolites extracted by ethyl acetate and chloroform against CRP1. DMSO was used as a control. Values are expressed as the mean ± SD (*n* = 3).

When tested by the agar well diffusion assay, it was found that in parallel to normal CFS, both the boiled CFS and proteinase K (PK)-treated CFS of CNE6 were also able to inhibit the growth of pathogen CRP1. Although the boiled CFS was able to produce a zone of inhibition, the zone diameter was slightly decreased in comparison to that of normal CFS (Fig. 6a).

### Production of hydrolytic enzymes by CNE6.

When endophytic strain *B. siamensis* CNE6 was grown on chitinase detection agar (CDA) plates, the strain was unable to produce any clear zone around its colonies. On the other hand, it produced a prominent yellow-colored zone (26 ± 1.5 mm) around bacterial growth on laminarin-containing plates, which were used to check the production of β-glucanase (Fig. 6c). In addition, isolated CNE6 also produced a clear halo zone (29 ± 1.7 mm) around bacterial growth on peptone gelatin agar (PGA) plates used for detecting protease enzyme production ability (Fig. 6f).

### Antifungal activity of crude extracts of CNE6 against CRP1.

Extracellular secondary metabolites in nutrient broth (NB) medium produced by CNE6 were extracted using different solvents, such as acetone, hexane, benzene, chloroform, and ethyl acetate (EA). In the agar well diffusion plates, among the crude extracts, only chloroform and EA extracts showed zones of inhibition against the pathogen CRP1 (Fig. 6h). This result suggested that only chloroform and ethyl acetate solvents were effective in extracting the antifungal metabolites produced in NB broth by CNE6. It was also observed that of these two active extracts, EA extract is more active against CRP1 than chloroform extract, based on the diameters of zones in agar well plates at the same concentrations.

### GC-MS analysis of active crude extracts.

For the detection of compounds present in chloroform and EA extracts of CNE6, gas chromatography-mass spectrometry (GS-MS) analysis was carried out. A total of five different compounds, namely, (E)-2-[1-(3-hydroxy 2furanyl) ethylidene]-(2H)-furan-3-one (CA), 6-azathymine, bis(methyl) ether (CB), 2,6-difluoro-3-methylbenzoic acid, 3-methylbutyl-2-ester (CC), 1,2,4,5-tetrafluorobenzene (CD), and (2E)-6-methoxy-2-[(4-methoxyphenyl)methylidene]-2,3-dihydro-1-benzofuran-3-one (CE), were identified with similarity indices of ≥90% from EA extract. In the chloroform extract, all compounds except (E)-2-[1-(3-hydroxy-2furanyl) ethylidene]-(2H)-furan-3-one were also detected after GC-MS analyses. In Table 2, the names, chemical formulas, and molecular weights of all the GC-MS-derived compounds of EA extract are depicted.

**TABLE 2:** Compounds derived after GC-MS analysis of crude ethyl acetate (no. 1 to 5) extract and chloroform extract (no. 2 to 5) of CNE6

No.	Compound name	Molecular formula	Mol wt	Peak area (%)
1	(E)-2-[1-(3-Hydroxy-2furanyl) ethylidene]-(2H)-furan-3-one	C_10_H_8_O_4_	192	32.74
2	6-Azathymine, bis(methyl) ether	C_6_H_9_N_3_O_2_	155	14
3	2,6-Difluoro-3-methylbenzoic acid, 3-methylbutyl-2-ester	C_13_H_16_F_2_O_2_	242	8
4	1,2,4,5-Tetrafluorobenzene	C_6_H_2_F_4_	150	26.69
5	(2E)-6-methoxy-2-[(4-methoxyphenyl)methylidene]-2,3-dihydro-1-benzofuran-3-one	C_17_H_14_O_4_	282	13.36

### Inhibition of biofilm formation of CRP1 by EA extract of CNE6.

**FIG 7:**
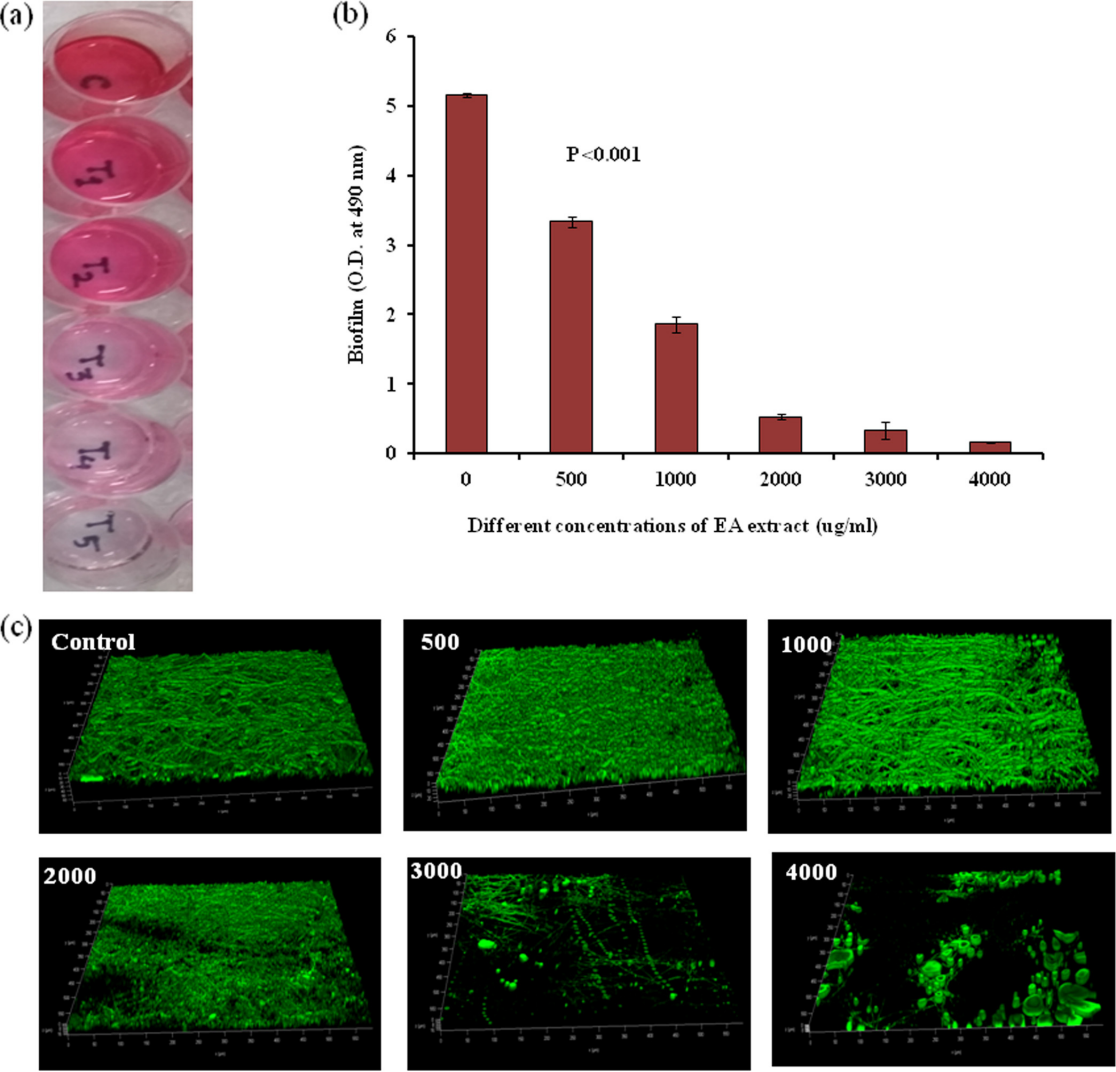
Inhibition of biofilm formation of F. solani CRP1 at different concentrations of EA extract of CNE6. (a) Biofilm observed in 24-well polystyrene cell culture plate after safranin staining; (b) quantification of biofilm formation at different concentrations of EA extract; (c) biofilm morphologies of F. solani CRP1 observed by CLSM after treatment. Values are expressed as the mean ± SD (*n* = 3), Significance level, *P* < 0.001.

Prominent biofilm formation by the isolated pathogen CRP1 was observed on polystyrene cell culture plates after 48 h of incubation at 28°C. EA extract of CNE6 was also effective for inhibiting the biofilm formation of the pathogen CRP1. Quantification of biofilm of F. solani CRP1 in different wells by use of a UV-Vis spectrophotometer strongly suggested a decrease (*P* < 0.001) in biofilm development along with increased concentrations of EA extract of strain CNE6 (Fig. 7a and b). Observations of the biofilm architecture in different treatment sets under a confocal laser electron microscope (CLSM) also strongly indicated that both filament thickness and substratum coverage area gradually decreased with increased concentrations of EA extract (Fig. 7c).

### Homology modeling.

Compounds derived after GC-MS analysis of EA extract were checked for activity against some of the particular targeted proteins of the pathogen F. solani CRP1. The structures of the target proteins were predicted using homology modeling for molecular docking studies. For this purpose, templates for lanosterol 14-alpha demethylase, beta-glucosidase, beta-tubulin, and squalene epoxidase were selected on the basis of similarity, and the templates used for such proteins were 6CR2, 5FJJ, 5SYC, and 6C6N (Protein Data Bank [PDB] IDs). The predicted structures were selected for molecular docking studies on the basis of MolProbity score, Ramachandran favored region, and Ramachandran outlier regions, respectively. The MolProbity score of the modeled proteins lanosterol 14-alpha demethylase, beta-glucosidase, beta-tubulin, and squalene epoxidase were 1.27, 1.52, 1.42, and 1.38, respectively, which were well suited for a protein. The Ramachandran favored regions of the proteins were found to be 93.23%, 92.01%, 97.13%, and 94.76%, respectively.

### Molecular docking.

**FIG 8:**
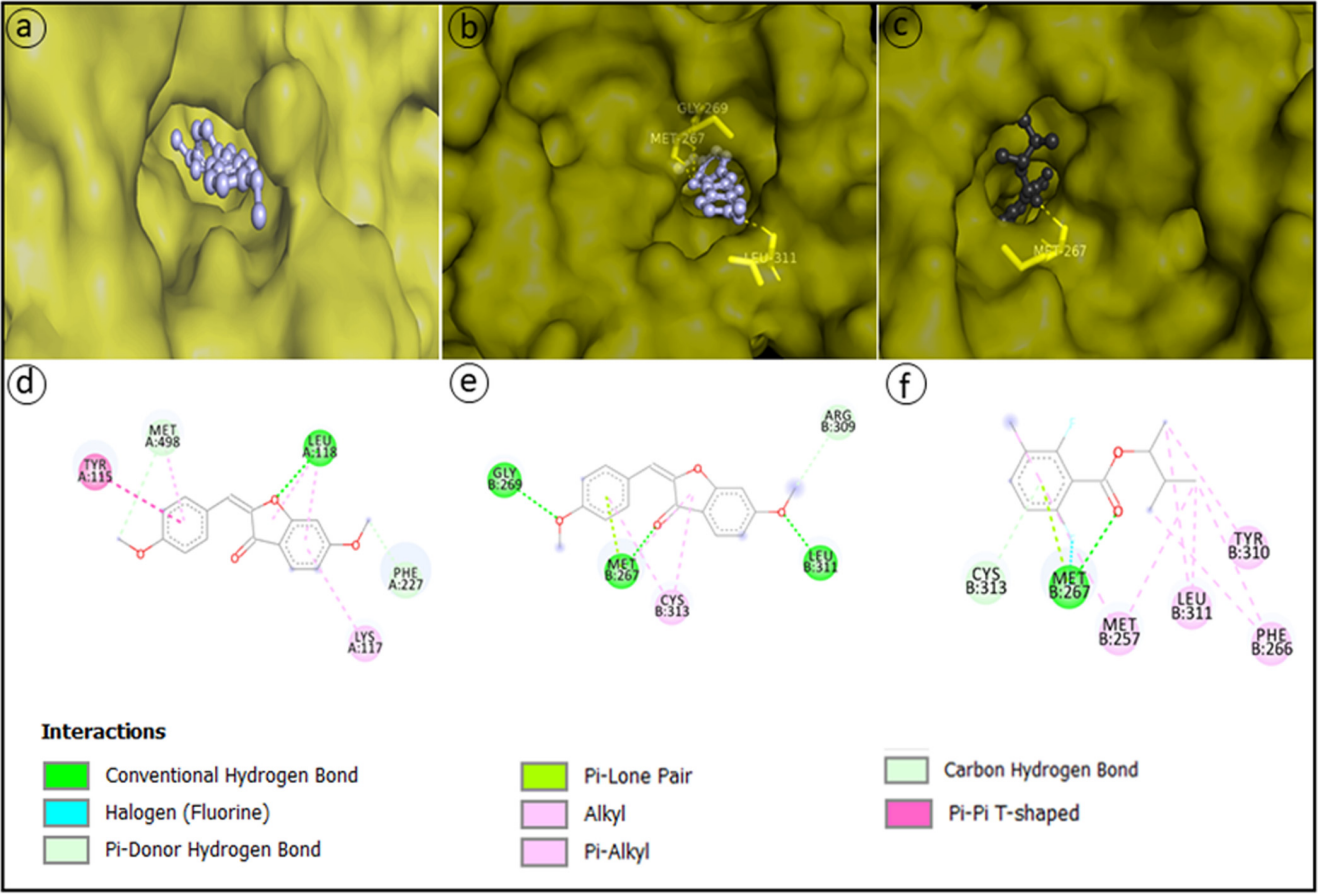
Molecular docking analysis showing interaction of the compounds. (a and d) 3D and 2D representations of docked complex of CE and lanosterol 14- alpha demethylase; (b and e) 3D and 2D representations of docked complex of CE and beta-tubulin; (c and f) 3D and 2D representations of docked complex of CC and beta-tubulin.

A molecular docking study was performed to determine the interaction between our active compounds and the four targeted proteins of the pathogen F. solani CRP1 and to determine which ligand binds effectively with which protein. The docked complexes (Fig. 8) were selected on the basis of free energy of binding and interactions of the ligand protein active residues. Molecular docking analysis showed that compounds CC (2,6-difluoro-3-methylbenzoic acid, 3-methylbutyl-2-ester) and CE [(2E)-6-methoxy-2-[(4-methoxyphenyl)methylidene]-2,3-dihydro-1-benzofuran-3-one] exhibited a good interaction with the beta-tubulin and free energy of binding values of −6.44 and −8.24 kcal/mol, respectively; the compound CE was also found to interact with the protein lanosterol 14-alpha demethylase, and the binding free energy was −7.52 kcal/mol. The binding free energy for the control tioconazole with lanosterol 14-alpha demethylase was −6.73 kcal/mol, and that of benomyl with beta-tubulin was −7.95 kcal/mol. The compound CE shows more negative binding energy than the control. From the visualization, it was found that CC formed one conventional H-bond and one lone-pair–π bond with the amino acid residue Met267, two alkyl bonds with Met257 and Leu311, and two π-alkyl bonds with Phe266 and one π-alkyl bind with Tyr310 of the protein beta-tubulin, and the compound CE also interacted strongly with the beta-tubulin and formed two hydrogen bonds with the amino acid residues Met267 and Leu311 and three π-alkyl bonds, two with Cys313 and one with Met267. The compound CE also inhibited the enzyme lanosterol 14-alpha demethylase, and the interacting amino residues were represented by Tyr115, Lys117, Leu118, Phe227, and Met498, respectively. All the interacting residues of the proteins lanosterol 14-alpha demethylase and beta-tubulin were present at the active pocket of the proteins. From the docking studies, we have also found that not a single compound was active against beta-glucosidase or squalene epoxidase.

### Effect of CNE6 in controlling black root rot disease of chickpea.

**FIG 9:**
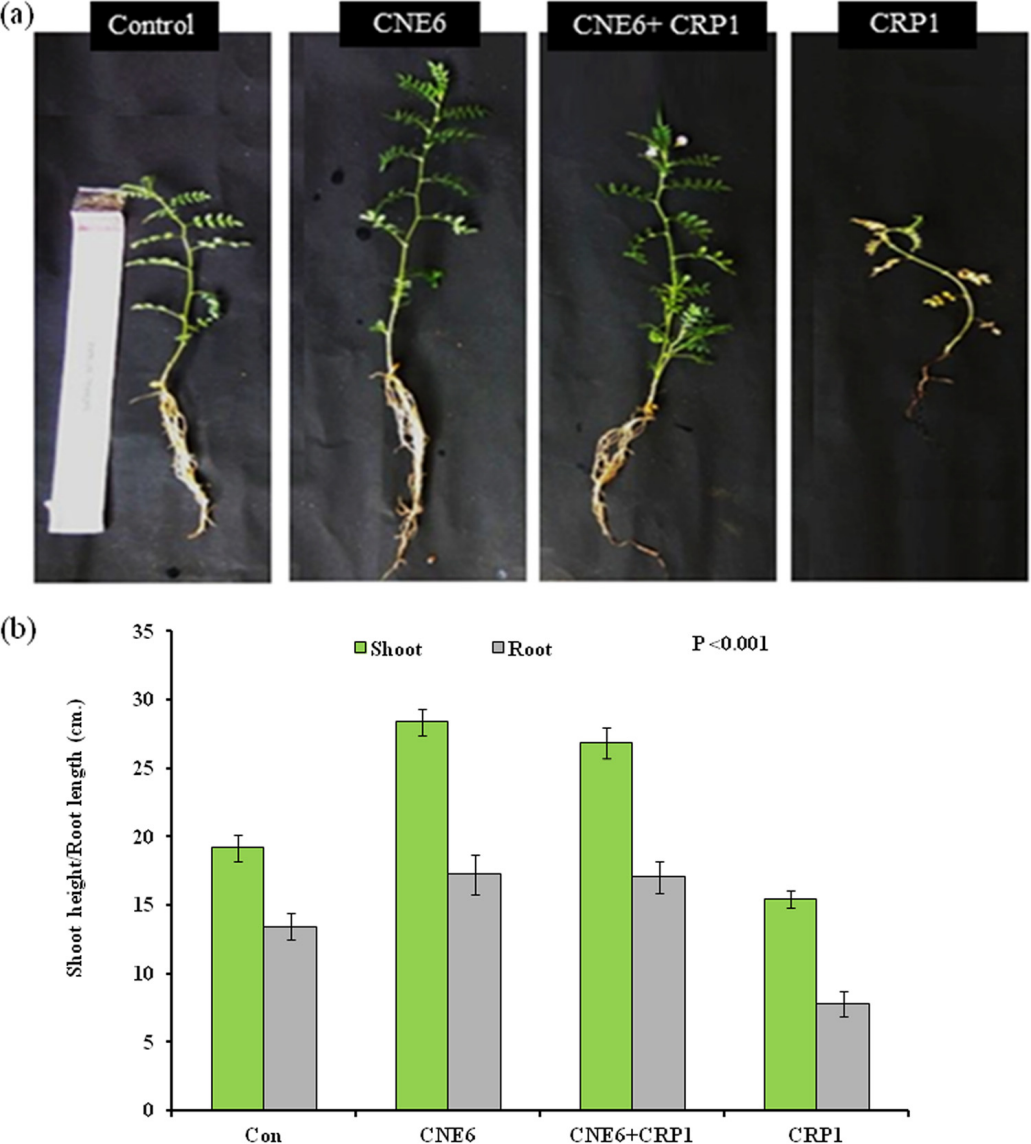
Effect of endophytic bacterium CNE6 in controlling black root disease and growth parameters (shoot and root length). (a) *In vivo* challenge experiment; (b) shoot and root lengths of different treated plants compared to those of untreated control set. Values are expressed as the mean ± SD (*n* = 6). Significance level, *P* < 0.001.

The antagonistic root nodule endophyte *B. siamensis* CNE6 was inoculated into different potted *Cicer arietinum* L. plants for the determination of its effect in suppressing black root rot disease caused by F. solani CRP1. At 25 days of growth of the chickpea plants, when the plants were observed for disease symptoms, it was found that plants treated with only the pathogen CRP1 were greatly affected while no disease symptoms were found in plants treated with CNE6 and CRP1. Rotten roots, black in color, and yellowing of leaves were observed in the plants of set 4, which were treated only with the pathogen CRP1. Significant increases in shoot height, root length, shoot biomass, and root biomass were noticed in plants treated only with CNE6 (set 2) in comparison to untreated control plants (set 1). On the other hand, plants treated with both CNE6 and CRP1 (set 3) also showed increased growth in terms of different growth parameters (Fig. 9a and b) in comparison to untreated control plants (set 1) and plants treated only with pathogen CRP1 (set 4). Although no visible symptoms were noticed in the plants of set 3, a slight decrease in growth was observed for set 3 in comparison to set 2, probably due to the stress generated by the pathogen in set 3.

### Expression of defensive genes of chickpea by CNE6.

**FIG 10:**
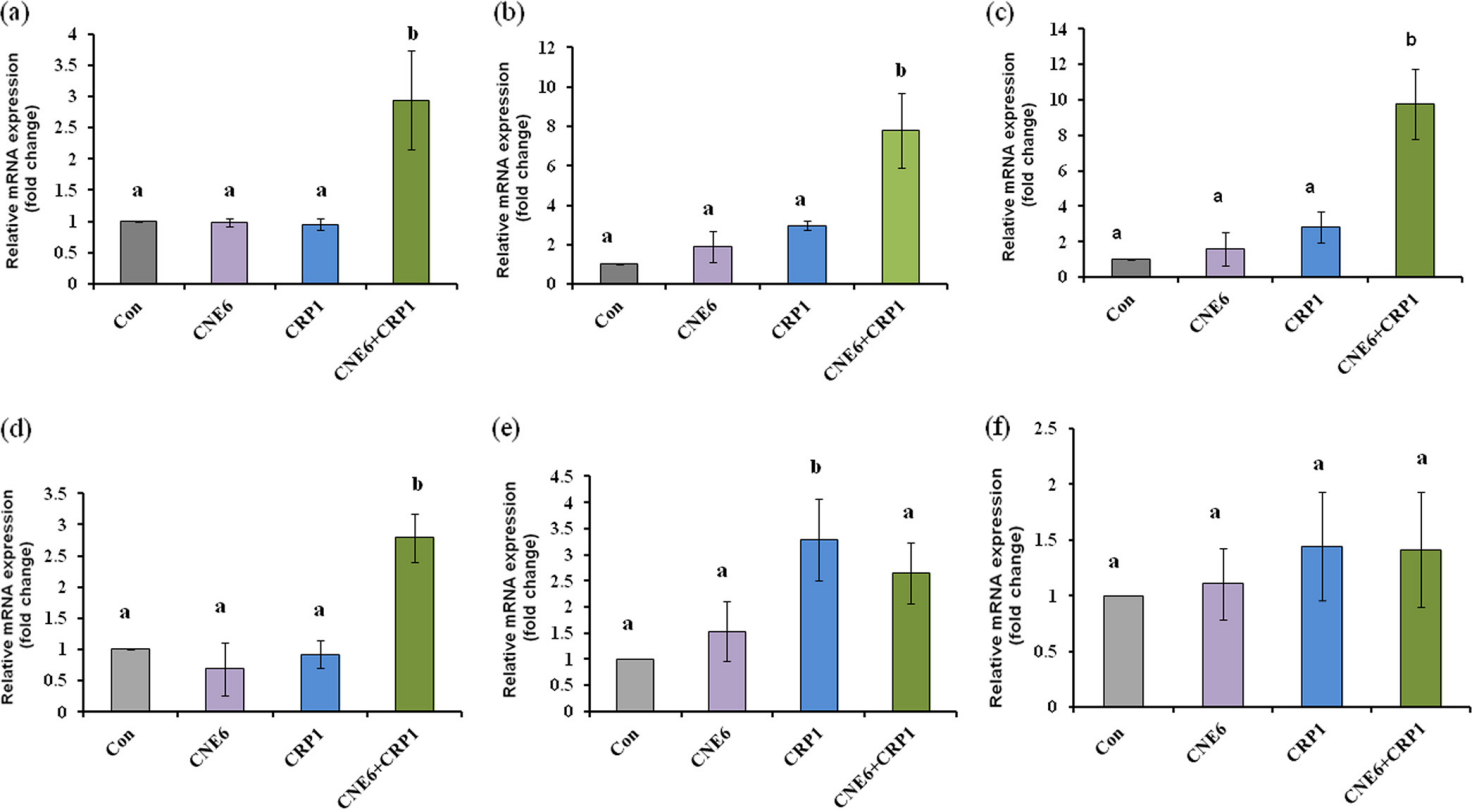
Ability of endophytic bacterium CNE6 to induce chickpea defense genes (expression of defense genes was monitored by qPCR analysis). (a) *CHI1*; (b) *PAMP*; (c) *PR2B*; (d) *TF1063*; (e) *TF1082*; (f) *PAL*. Values are expressed as the mean ± SD (*n* = 3). Means with different letters are significantly different. Significance level, *P* < 0.05. Con, control.

To assess the ability to induce the host defense system, the effect of the endophyte CNE6 on expression of chickpea genes related to antifungal activity, such as *CHI1* (chitinase I), *PR2B* (pathogenesis-related protein 2B), *PAMP* (polymorphic antigen membrane protein), *TF1063* (Myb, DNA-binding, homeodomain like), *TF1082* (pathogenesis-related transcriptional factor), and *PAL* (phenylalanine ammonia lyase), was observed. The expressions of all the above-mentioned genes were studied during a challenge experiment. During quantitative PCR (qPCR) analysis, significant upregulations of *CHI1*, *PAMP*, *PR2B*, and *TF1082* genes were observed when plants were treated with CNE6 and CRP1 (Fig. 10a to d). On the other hand, no significant increases in expression were noticed for the *TF1063* and *PAL* genes (Fig. 10e and f). It was also observed that neither the pathogen CRP1 nor the endophytic bacterial strain CNE6 was able to increase the expression of any of the genes significantly when they were applied separately, but when pathogen CRP1 and endophytic bacterium CNE6 were applied together, significant increases of the above-mentioned four genes were observed.

## DISCUSSION

Several scientists have found that chickpea plants are infected by various phytopathogens, and among them, the soilborne fungal pathogen Fusarium solani causing black root rot disease is one of the major threats in restricting chickpea production (3, 4). The pathogen F. solani has been previously identified as a causal agent for a number of crop diseases, such as fruit rot of *Cucurbita* spp., stem and root rot of Pisum sativum, dry rot of Solanum tuberosum, wilt disease of *Cicer arietinum*, and wilt sudden death syndrome of soybean (24). In Iran, Fusarium solani f. sp. *pisi* is also reported for black root rot disease of chickpea (24). The present study was focused on isolating the pathogen Fusarium solani causing black root rot disease in chickpea and management of this disease by using the previously isolated potent chickpea nodule endophytic bioagent *B. siamensis* CNE6 instead of chemical fungicides.

Since all of the isolated pathogenic strains showed similar morphological characteristics, one of the representative strains, CRP1, was selected for further studies. During the pathogenicity test, development of similar disease symptoms and reisolation of the pathogen from diseased plants, followed by molecular identification, confirmed Fusarium solani CRP1 as the pathogen of black root rot disease in chickpea plants grown in the Birbhum district, West Bengal, India. However, F. solani had already been identified previously by Nene et al. (2) as a causal agent of black root disease in chickpea.

In order to control the pathogen Fusarium solani CRP1, the endophytic bioagent CNE6 was used, and to understand the probable mechanisms, various experiments were conducted. In both the dual culture and agar well diffusion antagonism studies, the development of prominent zones signifies potential activity against the pathogen CRP1. The increased zone diameter (16.5 ± 1.5 mm) for 48-h-grown CFS indicated the secretion of more antifungal metabolites to the extracellular broth, especially during stationary phase of the culture. Hazarika et al. (25) demonstrated that CFS containing extracellular components of the endophyte Bacillus subtilis SCB-1 was able to inhibit a large number of phytopathogenic fungal growths on potato dextrose agar plates.

Measurement of fungal radial growth is a well-known but indirect method for determining the antifungal potential of any chemicals or metabolites (26). Several biocontrol organisms have been reported to inhibit the radial growth of pathogenic fungi when tested *in vitro*. For example, the 1-butanol extract of B. subtilis 30VD-1 at a 100-μg/mL concentration was found effective for 40% inhibition of radial growth of Fusarium sp. (27). Antifungal metabolites produced by endophytic CNE6 inhibit the radial growth of CRP1. Therefore, application of CNE6 will be helpful in suppressing pathogenic growth and its spread within the host tissues.

Conidia of Fusarium spp. can survive for a long time in soil (28). Successful germination of conidia in the soil plays a significant role in disease development of cultivated crops. Therefore, inhibition of conidial germination and germ tube elongation is the best way to protect the crop plants from fungal phytopathogenic attack. Metabolites produced by CNE6 have strong activity to suppress the germination of conidia of the pathogen Fusarium solani CRP1. So, our results suggested that CNE6 will definitely play an important role in reducing disease severity. Morphological aberrations of CRP1, especially the degradation of cell wall structures, suggested that strain CNE6 may produce cell wall-degrading enzymes like chitinase, β-glucanase, and protease. The primary components of cell walls of true fungal cells are chitin and β-glucan, along with some glycoproteins. For the inhibition of fungal phytopathogens, the production of chitinase and β-glucanase enzymes was considered an especially significant characteristic of a biocontrol agent (8). Some groups of researchers have also postulated that due to the presence of proteolytic enzymes such as protease or trypsin, the activity of other fungal cell wall-degrading enzymes was significantly increased and the release of glucose and *N*-acetyl-d-glucosamine was stimulated (29). It was recorded earlier that Bacillus velezensis NKG-2 was able to produce hydrolytic enzymes related to fungal cell wall degradation and was effective in inhibiting phytopathogens (7). Another report was also published regarding *Bacillus* spp. with protease-encoding genes having antagonistic activity against the pathogen Fusarium sp. (27). An *in vitro* test of CNE6 regarding protease and β-glucanase production strongly supports the positive results in these two cases.

Thi et al. (30) observed that the chitin binding protein of 22 kDa produced by Bacillus subtilis XL62 showed very good antifungal potential even after treatment with PK (0.5 to 2.5 μg/mL) and after boiling at 100°C for 60 min. To understand the nature of antifungal metabolites produced by CNE6, such types of tests are used and good levels of antifungal activities are observed after heat or proteinase K treatment of CFS of CNE6. Retention of zones after boiling the CFS at 100°C for 10 min and after PK treatment suggested that the antifungal metabolites produced by CNE6 are thermostable and nonproteinaceous or that they may be insensitive to PK. Antifungal activity was slightly reduced by such treatment in comparison to that of untreated CFS of CNE6. This may be due to the production of both proteinaceous and nonprotein antifungal metabolites by the isolates or may be due to little degradation of proteinaceous antifungal metabolites after treatment. Positive results for β-glucanase and protease enzyme production are also supported by the proteinaceous antifungal metabolite production by CNE6. To extract the antifungal metabolites produced by CNE6, a solvent extraction process was also followed. Among the different solvents used, EA and chloroform were found effective for extracting the antifungal metabolites from the CFS. Earlier, several endophytic and nonendophytic *Bacillus* spp., including Bacillus siamensis, were reported to produce a wide variety of structurally different antagonistic substances as their secondary metabolites (31). The EA fraction of *B. velezensis* Lle-9 contained various secondary metabolites, such as cyclopeptides, linear peptides, and some antibiotics, etc., which were reported to have antifungal potential (32). A strain of B. subtilis C9 and its EA fraction were purified, and the antibiotic substances that were extracted had antagonistic activity against phytopathogenic fungi (33).

Researchers have found that benzofuran and its derivatives are widely present in several natural and unnatural products that show a wide range of pharmaceutical and biological potential (34). Our data recorded from GC-MS results of the CNE6 extract indicated five different compounds, which have already been mentioned in Table 2. EA and chloroform extract showed almost the same results during GC-MS analysis, but better activity was observed for EA extract, which may be due to the presence of one extra compound or to the presence of another four compounds at higher concentrations than in the chloroform extract.

One of the virulence factors of most pathogenic microorganisms is the formation of biofilm in the particular habitat during the infection process. Inhibition of biofilm formation of the pathogenic microorganisms is regarded as one of the control measurement techniques which may reduce their virulence properties or make them susceptible to antimicrobial substances. Formation of biofilm by F. solani CRP1 was almost completely inhibited at EA fraction concentrations of 4,000 μg/mL, where no fungal filament was found to develop on the substratum. A molecular docking study revealed that compound CE [(2E)-6-methoxy-2-[(4-methoxyphenyl)methylidene]-2,3-dihydro-1-benzofuran-3-one] was found to be the most active of the five compounds against the proteins beta-tubulin and lanosterol 14-alpha demethylase. As a result, compound CE interferes in the assembly of microtubules and also inhibits the activity of the enzyme lanosterol 14-alpha demethylase.

Not only does the endophytic bacterium help the plants by directly inhibiting the phytopathogen, but it also might be effective in the indirect induction of some host defensive genes against the phytopathogens. An *in vivo* challenge study for understanding defensive gene expression as well as growth enhancement of chickpea suggested that the endophyte CNE6 plays positive roles in triggering plant antifungal defense-related genes, especially *CHI1*, *PAMP*, *PR2B*, and *TF1082*. The production of several proteins in chickpea by the genes *PR2B*, *PAMP*, *TF1082*, and *TF1063* in response to a fungal pathogenic attack has already been reported by Leo et al. (35). The gene *PAL* that is involved in the phenylpropanoid pathway was also found to be induced in chickpea plants against Ascochyta rabiei (36). When the endophyte CNE6 was cultured in CDA plates containing soluble chitin, no prominent zones were observed, but here, significantly, CNE6 was effective in inducing the host plant’s chitinase production, which probably occurs due to some secondary metabolites that were synthesized during a tritrophic (host-CNE6-CRP1) interaction. A significant increase in the expression of defensive genes in the presence of the pathogen and the biocontrol agent together may be due to the production of some molecules by the endophytic bacterium CNE6 in the presence of the pathogen that induces the host plant’s defense. Gond et al. (20) also found that endophytic *Bacillus* spp. were able to induce host plant (maize) defensive genes like *PR-1* and *PR-4* for suppressing fungal phytopathogens. It was also reported earlier that the endophytes B. subtilis and *B. amyloliquefaciens* stimulate antifungal defensive genes like *SOD-2*, *PR-1*, and *PR-4* in maize seedlings (9). Scientists have described that most of the plants during fungal pathogenic infection synthesize different PR proteins like β-1,3-glucanase, which breaks the fungal cell wall material glucan as well as glycoproteins and peptides (37, 38). Another PR protein, chitinase, was also found to be synthesized against fungal phytopathogens. Leo et al. (35) observed that in various cultivars of chickpea, a number of genes are expressed at different levels to control the pathogen *Ascochyta rabiei*.

From these observations, it is clear that the isolate *B. siamensis* CNE6, which was previously reported as an endophyte from chickpea plants (22), has a promising role in preventing pathogenic infection and for inducing the growth of chickpea plants even in the presence of the pathogen F. solani CRP1. The endophyte CNE6 can play a significant role in increasing chickpea growth and development by suppressing the pathogen CRP1, which causes black root disease in chickpea plants under natural environmental conditions.

### Conclusion.

The present investigation confirmed F. solani CRP1 as the pathogen of black root rot disease of chickpea grown in West Bengal, India, and the endophytic strain *B. siamensis* CNE6 showed sufficient antifungal potential against the isolated pathogen. CNE6 is not only involved in the direct inhibition of the pathogen by producing different types of antifungal metabolites but also increases the host immunity against the pathogen by inducing the host plant defense system. An *in vivo* challenge experiment also established the potential of CNE6 in combating disease symptoms even in the presence of high pathogenic loads. Based on overall observations, the endophytic strain CNE6 can be considered a prospective biocontrol agent for controlling black root rot disease in an eco-friendly manner.

## MATERIALS AND METHODS

### Collection of plant samples.

*Cicer arietinum* L. plants infected with black root rot disease were collected from a farmer’s field located at Nanoor, Birbhum (23°35’20’’N and 87°20’25’’E), of West Bengal, India. During sample collection, the plants were uprooted carefully and bound soil particles were removed with a little shaking. Samples were then taken into the laboratory in sterilized polyethylene bags. After proper washing, samples were subjected to isolation of the fungal pathogen causing black root rot disease.

### Isolation and characterization of pathogen causing black root rot disease of chickpea.

Initially, the infected parts of roots were separated and immersed for 2 min into 0.1% HgCl_2_ solution for the surface sterilization. Finally, the samples were rinsed four times with sterilized distilled water for removal of HgCl_2_. Small pieces of diseased roots were placed on malt extract (ME; Himedia, India) agar plates amended with streptomycin (200 μg/mL) (HiMedia, India) and incubated at 28°C for 72 h (39). After the emergence of fungal mycelia from the infected regions, the mycelia were purified by streaking on ME agar plates. Purified colonies were maintained after repeated subculturing on ME agar slants and kept in a refrigerator at 4°C.

The morphological characteristics of the isolated fungal pathogens were visualized under a light microscope (Zeiss Primostar) after staining with cotton blue and lactophenol on a glass slide.

### Identification of isolated pathogens.

One of the representatives of the isolated fungal pathogen CRP1 was then identified based on molecular characteristics. For this, genomic DNA of CRP1 was extracted with the help of a HiPurA fungal DNA purification kit (MB543; HiMedia, India) and PCR amplification of internal transcribed spacer (ITS) regions was carried out by using ITS1/ITS4 primers (40). The amplified DNA was resolved on a 1.2% agarose gel and then extracted and purified by using an XcelGen DNA gel/PCR purification miniprep kit (Xcelris, India). Reactions of DNA sequencing were carried out using a BigDye Terminator v3.1 cycle sequencing kit on an ABI 3730xl genetic analyzer. Finally, nucleotide sequences were used for BLAST in NCBI GenBank (https://www.ncbi.nlm.nih.gov/). On the basis of the maximum identity score, the first 15 sequences were taken and aligned with the help of ClustalW. Kimura’s two-parameter model (41) was followed for computing the distance matrix, and the neighbor joining (42) phylogenetic tree was constructed by using MEGA 6 (43).

### Pathogenicity test.

The isolated pathogen was tested for its ability to develop black root rot disease in chickpea seedlings. Seeds of chickpea were surface sterilized in accordance with the previously described method (see above) and were allowed to germinate at 25°C for 72 h by keeping them in sterilized petri plates containing sufficient water-soaked blotting paper. Then, the study was performed in the following two ways.

### (i) *In vitro* laboratory assessment.

In the *in vitro* laboratory assessment, after the development of roots, sterilized phosphate buffer saline (PBS) water-suspended conidia (5.5 × 10^4^ conidia/mL) of CRP1 were applied directly to the petri plates (*n* = 3) containing germinated seeds, and this was considered the treated set; the conidia were again allowed to grow for another 5 days to observe disease symptoms in the developing roots. In the control set (*n* = 3), only PBS was applied.

### (ii) *In vivo* pot experiment.

An *in vivo* pot experiment was also performed to confirm the isolated strain CRP1 as the black root rot pathogen of chickpea. A total of 12 plastic pots were prepared containing sterilized soils and vermicompost (3:1, vol/vol) and divided into two groups (*n* = 6). Seeds germinated for 3 days were then planted in pots at a 1-cm depth in the soil. The pot experiment was performed in an open environment. Twelve days after the seedlings were planted, conidia (5.5 × 10^4^ conidia/mL) of pathogen CRP1 suspended in PBS were sprayed on the soils of the respective pots (treatment set). The control set was treated with only PBS. After 30 days, plants from both sets were uprooted and checked for the development of black root rot disease symptoms. Finally, the pathogen was again reisolated and identified through molecular characteristics.

### Biocontrol agent used for controlling the pathogen.

One Gram-positive endophytic bacterial strain, Bacillus siamensis CNE6 (GenBank accession no. MT032484.1), isolated from root nodules of *Cicer arietinum* (22) was used for the present study. The strain was selected due to its multifaceted plant growth-promoting attributes, including broad-range antifungal potential and very good root-colonizing potential (22).

### Antagonistic activities of CNE6 against the isolated pathogen CRP1. (i) Dual culture overlay assay.

*In vitro* antagonistic activity of the biocontrol strain CNE6 against the isolated pathogen CRP1 was assessed by using the dual culture overlay method (23). In this method, a single line of the endophytic bacterial strain CNE6 was inoculated onto nutrient agar (NA) plates and incubated at 28°C. After 48 h of bacterial growth, 100 μL of conidial suspension (2.2 × 10^4^ conidia/mL) of CRP1 was mixed with 5 mL of ME soft agar (0.7% agar agar) and overlaid on NA (HiMedia, India) plates containing CNE6 culture. Zones of inhibition were recorded after 72 h of incubation at 28°C.

### (ii) Agar well diffusion assay.

Antifungal activity of the selected strain CNE6 was also tested by the agar well diffusion method (44). To perform this experiment, cell-free supernatant (CFS) of strain CNE6 was collected from 24-h- and 48-h-grown culture. CFS was prepared by centrifugation at 10,000 rpm for 15 min, followed by membrane filtration using a 0.22-μm cellulose acetate membrane. One hundred microliters of CRP1 conidial suspension (2.2 × 10^4^ conidia/mL) was sprayed on an ME agar plate, and then wells were made in the agar plate using a cork borer. Fifty microliters of each CFS (collected from 24- and 48-h-grown culture) of CNE6 was added to these wells and incubated for 72 h at 28°C. Zones of inhibition around the wells were measured. Wells with only NB medium were considered the untreated control.

### Study of inhibition of radial growth of CRP1 by CFS of CNE6.

The ability of CFS of CNE6 to inhibit radial growth of the pathogen CRP1 was also studied (45). CFS was collected from the 48-h-grown culture of CNE6. CFS, 10 times concentrated, was mixed at different percentages (0% to 50%) with the ME plates before solidification. After solidification, the plates were then inoculated with an agar disk (5-mm diameter) containing pathogenic fungal mycelia. After 7 days of incubation at 28°C, the radial growth in both the control (C) and treated (T) sets was measured. The percentage of radial growth inhibition (*I*) was calculated by the formula: *I* (%) = [(*C* − *T*)/*C*] × 100. The corrected inhibition (*IC*) was then calculated as follows: *IC* (%) = [(*C* − *T*)/(*C* − *C*_0_)] × 100, where *C* is the diameter of a fungal colony in control plates, *T* is the diameter of a colony in treated plates, and *C*_0_ is the diameter of the pathogenic fungus agar disk (5 mm).

### Determination of effects of CFS on conidial germination of CRP1.

For the conidial germination assay, equal volumes of conidial suspension in PBS (40 to 50 conidia per 10× microscopic field) and CFS of CNE6 at different percentages (0% to 50%) were mixed in cavity slides and the slides were incubated overnight at 28°C under moist conditions (46). In the control set, only sterilized NB was mixed with the conidial suspension. The germination patterns were observed under a light microscope (Zeiss Primostar), and the percentages of conidial germination were calculated by the following formula: (number of germinated conidia/total number of conidia) × 100.

### SEM study.

The SEM study was performed to observe the effects of extracellular secondary metabolites of CNE6 on the cellular morphology of the isolated pathogen CRP1. Mycelia of the phytopathogenic strain CRP1 were taken from the edge of inhibition zones produced in dual culture plates and were considered the treated mycelia. Alternatively, mycelia taken from a freshly grown culture of CRP1 on ME agar plates were regarded as the control. Mycelia were prefixed for 30 min using 2% glutaraldehyde in 20 mM Na-P buffer (pH 6.5) plus 5% dimethyl sulfoxide (DMSO). Then, samples were postfixed for another 30 min using 1% osmium tetroxide dissolved in 50 mM Na-P buffer (pH 6.5). After washing with sterilized distilled water, the mycelia were dehydrated by passage through a series of alcohol grades (30 to 100%) and by retaining them at least 10 min in each grade (47). The dehydrated mycelia were then gold coated by using an ion sputter (Quorum sputter coater, model SC7620) and observed through the microscope (Zeiss; GeminiSEM 450).

### Determination of the nature of antifungal metabolites.

The thermostability of the antifungal metabolites produced by the endophyte Bacillus siamensis CNE6 was studied by keeping the CFS in a boiling water bath for 10 min. In addition, to check the proteinaceous nature of the antifungal principles, CFS was treated with proteinase K (1 mg/mL) (HiMedia, India) for 2 h at 37°C (48). The antifungal potential of both heat-killed and proteinase K-treated CFS was studied against the isolated pathogen CRP1 by using the agar well diffusion method (44). Untreated CFS was used as the control.

### Study of hydrolytic enzyme production by isolate CNE6.

The ability of CNE6 to produce fungal cell wall-degrading enzymes and the production of chitinase, β-glucanase, and protease by the strain were studied. Chitinase activity of strain CNE6 was tested by following the conventional plate method using chitinase detection agar (CDA) (49). For this, M9 medium with additions of 1% colloidal chitin (HiMedia, India) and 2% agar agar was used (HiMedia, India). Colloidal chitin was prepared by following the protocol of Berger and Reynolds (50). A 10-μL drop of fresh CNE6 bacterial culture was added to the CDA plate, incubated at 28°C for 7 days, and regularly checked for the formation of a clear solubilization zone around the bacterial growth.

For β-glucanase activity, CNE6 was inoculated onto the plate containing mineral minimal medium supplemented with 0.5% laminarin (Sigma-Aldrich, India). After 72 h of incubation (28°C), the plates were flooded with Congo red (0.6 g/L) and kept for 90 min at room temperature. Excessive stains were poured out and checked for hydrolysis of glucan by detecting the yellow or orange zone around the bacterial growth (51).

In addition, protease activity was checked in a peptone gelatin agar (PGA) plate. PGA medium (pH 7) contains peptone (5 g/L), gelatin (4 g/L), and beef extract (3 g/L) (Merck, India), amended with 2% agar agar. Fresh bacterial inoculum (optical density at 620 nm [OD], 0.5) was spotted onto the PGA plate and incubated for 24 h at 28°C. To confirm the proteolytic activity of the strain, the bacterial plate was flooded with 15% HgCl_2_ (52). Strain CNE3 and Bacillus velezensis SEB1 were used as negative and positive controls, respectively, for both the β-glucanase and protease tests.

### Extraction of antifungal metabolites produced by CNE6.

To extract the extracellular antifungal compounds produced by strain CNE6, bacteria were grown in NB broth with mild shaking (120 rpm) at 28°C. After 72 h of growth, the culture was centrifuged at 10,000 rpm for 15 min and CFS was collected. The CFS was then filtered using a 0.22-μm cellulose acetate membrane. Metabolites were extracted from the CFS with the help of a separating funnel by using different organic solvents like acetone, benzene, chloroform, diethyl ether, and ethyl acetate (EA) at a 60:40 (vol/vol) ratio. All the fractions were collected and left in a vacuum centrifugal evaporator for drying. The dried material was redissolved in sterilized DMSO at a concentration of 1,000 μg/mL, and antagonistic activity against CRP1 was checked by observing inhibition zones in accordance with the agar well diffusion method (44). The most active fraction was then used for further studies.

### Study of biofilm inhibition of CRP1 by EA extract of CNE6. (i) Determination of biofilm formation ability of the pathogen CRP1.

In accordance with the method of Imamura et al. (53), the biofilm-forming ability of the fungal pathogen CRP1 under *in vitro* conditions was studied. For this, 1 mL of ME broth containing conidia of CRP1 (10^4^/mL) was added to the wells of 24-well flat-bottom polystyrene cell culture plates and incubated for 48 h at 28°C. After that, nonadherent fungal cells were washed gently with PBS. Uninoculated ME broth was considered the control. Fungal cells attached to the plastic surfaces were confirmed as true biofilm formation. All the experiments were done in six replicates.

### (ii) Inhibition of biofilm formation of CRP1 by EA fraction of CNE6.

Inhibition of biofilm formation of the fungal pathogen CRP1 by EA extract of CNE6 was checked in 24-well polystyrene cell culture plates. Similar to the previous description, fungal conidia were inoculated in 1 mL of ME broth and then added to each well. EA extract of CNE6 dissolved in 10% sterilized DMSO (100 μL) was added at different concentrations (0 to 1,000 μg/mL) to different wells and incubated for 24 h at 28°C. After that, broth was removed from each well and the wells were carefully washed twice with PBS. Biofilms were fixed with 95% ethanol for 15 min at 37°C and finally stained with 1 mL of 0.1% safranin (54) and left for 5 min. Excessive safranin was eluted from the wells by washing with sterilized distilled water and air dried again. To extract the bound safranin from the fungal cells, 1 mL of 30% glacial acetic acid was added to each well and left at room temperature for 5 min. Color changes of the acetic acid solution were measured at 490 nm with the help of a UV-Vis spectrophotometer (Shimadzu; UV-1700).

### CLSM study.

The architecture of CRP1 biofilms was analyzed after treatment with different concentrations of EA extract using a confocal laser scanning microscope (CLSM) (Leica TCS SP8). For this purpose, before the addition of fungal conidia as well as EA fraction, sterilized cover glasses (1 cm by 1 cm) were placed in each well. After biofilm development, the cover glasses were carefully removed and nonadherent cells were washed with PBS. Finally, the structure of the biofilm was observed under the CLSM (excitation wavelength, 488 nm) after staining with 0.1% (wt/vol) acridine orange (55).

### GC-MS analysis of crude active fractions of CNE6.

In order to determine the presence of antifungal compounds, chloroform and EA extracts were subjected to GC-MS analysis. For this, ion trap technology was followed by using a TR-Wax GC column (Thermo Fisher Scientific). An initial temperature of 50°C for 2 min and a final temperature of 280°C were maintained in the instrument. At a flow rate of 1 mL/min, helium gas was used as the carrier and a 2-μL volume of sample was injected (spit ratio, 10:1) into the injection port by maintaining the temperature at 250°C. An ionizing voltage of 70 eV was used for mass spectra. The compounds present in the crude EA extract were identified with the help of similar mass spectra of the compounds present in NIST05 (National Institute of Standards and Technology, USA) library data.

### Ligand-based molecular docking study against the phytopathogen F. solani: prediction of drug target proteins of F. solani using homology modeling.

Compounds identified after GC-MS analysis were subjected to molecular docking analysis against the four targeted proteins of the pathogen F. solani. As the crystal structure of the target proteins of F. solani were not available in the PDB (https://www.rcsb.org/), the initial homology modeling study was performed for the prediction of different protein structures. At first, the protein sequences for lanosterol 14-alpha demethylase, beta-glucosidase, beta-tubulin, and squalene epoxidase were retrieved from UniProt (https://www.uniprot.org/), and then they were used to search for similarity with other organisms using the Basic Local Alignment Search Tool (BLAST) in the NCBI data bank against available PDB proteins. The templates for homology modeling were selected on the basis of similarity from BLAST results.

For the prediction of protein structures through homology modeling, the SWISS-MODEL server (https://swissmodel.expasy.org/) (56) was used. For the evaluation and assessment of the Ramachandran plot of the modeled proteins lanosterol 14-alpha demethylase, beta-glucosidase, beta-tubulin, and squalene epoxidase, we subjected the models to MolProbity analysis in the structure assessment tool of the SWISS-MODEL web server. The validated model proteins were selected for the molecular docking study. A comparative study of the model proteins was performed by the PyMOL visualizing tool (The PyMOL Molecular Graphics System, version 2.0; Schrödinger, LLC).

### Molecular docking analysis.

Compounds derived after GC-MS analysis were checked for activity against the particular targeted proteins of F. solani CRP1. AutoDock 4.2 of the AutoDockTools 1.5.6 molecular docking program was used for molecular docking studies. Tioconazole and benomyl were used as controls against lanosterol 14-alpha demethylase and beta-tubulin. The preparation of the three-dimensional (3D) format of all the ligands was done using ChemDraw, and optimization of the ligands was performed using PM6 of a semiempirical method in GaussView 5.0. The optimized ligands were then used to perform molecular docking analysis. The active sites of the proteins were predicted using CASTp (57). In addition, structures of the modeled proteins were prepared by using AutoDockTools for molecular docking studies. At first, the polar hydrogens were added to the proteins, Kollman charges were then added and Gasteiger was computed, and finally, the AD4 type was assigned. For AutoDock 4.2, a grid box that was sufficiently large to cover the entire protein binding site and allow the ligand to move freely was generated. The dimension sizes of the grid along *x*, *y*, and *z* axes were 80 by 70 by 68 Å, 90 by 70 by 90 Å, 120 by 80 by 70 Å, and 80 by 110 by 80 Å for lanosterol 14-alpha demethylase, beta-glucosidase, beta-tubulin, and squalene epoxidase, respectively, and the distances between two connecting grid points were 0.653 Å for lanosterol 14-alpha demethylase and 0.375 Å for beta-glucosidase, beta-tubulin, and squalene epoxidase. Lamarckian genetic algorithm (LGA) was used in AutoDock4 (58) for receptor-fixed ligand-flexible docking calculations to enhance performance in comparison to simulated annealing. The maximum number of generations of the LGA run before termination was 27,000, and the maximum number of energy evaluations before the termination of LGA run was 2,500,000. Software’s default values were set for other docking parameters. For each protein-ligand docking, a maximum of 10 binding poses were generated, and the most suitable pose was chosen on the basis of the interaction of the active-site amino acid residues with ligand. Visualization and analysis of the docked complex of protein and ligand were done by PyMOL and Discovery Studio Visualizer of Accelrys Discovery Studio (https://discover.3ds.com/discovery-studio-visualizer-download).

### *In vivo* challenge experiment.

An *in vivo* challenge experiment was performed to detect the roles of isolated strain CNE6 for increasing chickpea growth as well as for inducing the defensive genes upon challenge with the pathogen F. solani CRP1. Three-day-old germinated seeds were planted below 1 cm of soil in four different sets (*n* = 6). Before planting, seedlings of set 2 and set 3 were treated for 2 h by dipping the roots in PBS containing a bacterial suspension of CNE6 (OD_620_, 0.5), while seedlings of set 1 and set 4 were dipped into only PBS. All the plants were allowed to grow in a natural environment. Ten days after the planting of seedlings, a conidial suspension of the pathogen CRP1 was sprayed into the soil of set 3 and set 4. Plants were watered as required. After 12 days of pathogen treatment, the shoot height, root height, total biomass, and disease symptoms of the plants were recorded. In addition, leaf tissues of each set were collected for RNA extraction.

### Study of defensive gene expression of chickpea.

In order to determine the level of expression of some antifungal defensive genes, namely, *CHI1* (chitinase I), *PR2B* (pathogenesis-related protein 2B), *PAMP* (polymorphic antigen membrane protein), *TF1063* (myb, DNA-binding, homeodomain like), *TF1082* (pathogenesis-related transcriptional factor), and *PAL* (phenylalanine ammonia lyase), of chickpea by the endophyte CNE6 in the presence of Fusarium solani CRP1, tissues of chickpea were collected separately from all the sets of the *in vivo* experiment and carefully brought into the laboratory for RNA extraction. One hundred milligrams of tissues from chickpea plants from each set was collected, and total RNA was extracted using an RNeasy plant minikit (Qiagen, India) by following the manufacturer’s protocol. The purity and concentration of total RNA were quantified with the help of a NanoDrop ND1000 spectrophotometer. cDNA was prepared according to the manufacturer’s protocol of RevertAid H minus first strand cDNA synthesis kit (Thermo Fisher Scientific). qPCR was performed by using a Quant Studio TM3 cycler (Applied Biosystems). Primers used for qPCR are provided in Table 3 (35, 36, 59–62). All the PCRs were performed by following the Go Taq qPCR master mix standard protocol of Promega. Expression of the defensive genes was analyzed according to the method of Schmittgen and Livak (63).

**TABLE 3:** Primer sets used during qPCR analysis of chickpea defensive genes

Gene ID	Gene name	Function	Primers	Reference
EU529707	*ACT1*	Housekeeping gene	F-GCCTGATGGACAGGTGATCAC	35
			R-GGAACAGGACCTCTGGACATCT	
	*CHI1*	Hydrolysis of chitin (main component of fungal cell wall)	F-AAGGCTTTGGTGCAACCATT	Designed by using Primer3 v.0.4.0 (36)
			R-CACCAGGAGCAACACCCAAT	
CV793598	*PR2B*	β-1,3-Glucanase—hydrolysis of flavonoid and isoflavonoid compounds	F-GCCTAGAAAGGCAAATCCTTC	37
			R-CATCTGCCGTGGGAATAAGA	
DY475248	*PAMP*	Transcription of defense-related genes—resistance response via metabolism utilizing polyamines and nicotianamines	F-CCGCTGATACAGTGGAGGTT	38
			R-GTTTCCCCAATTTCCTCACC	
TF1070.m00005	*TF1063*	Host defense gene (QTL_AR1_–LG4)	F-GTTATGTGGGTGGAGTTGGAA	39
			R-CAACCATAGCTGCAACCATCT	
TC101530	*TF1082*	Host defense gene (QTL_AR2_–LG4)	F-AAGTCTTATCGTGGCGTTCG	38
			R-TCATAAGCTAGTGCTGCTGCT	
	*PAL*	Induced phenyl propanoid pathway	F-AAGCTCACTTGCAGAGACAT R-ACCTGATGCAGTGATTGTGC	40

### Statistical analysis.

Data from each experiment are represented as the mean value of at least three replicates. Means and standard deviations (SDs) were calculated by using Microsoft Excel Program version 2010. In order to study the significant (*P* < 0.05) difference between control and test samples of the respective studied parameters, a single-factor analysis of variance (ANOVA) test was performed. Only data of qPCR were analyzed through variance analysis (ANOVA test) with repeated measurements (*n* = 3) using the Tukey method in Minitab 17 software for testing the significant differences (*P* < 0.05) among the control and treatment sets. Fisher’s least significant differences (LSD) were also calculated in the respective results.
